# A novel nonsense variant (c.1499C>G) in *CRB1* caused Leber congenital amaurosis-8 in a Chinese family and a literature review

**DOI:** 10.1186/s12920-022-01356-z

**Published:** 2022-09-17

**Authors:** Wenhua Duan, Taicheng Zhou, Huawei Jiang, Minhui Zhang, Min Hu, Liwei Zhang

**Affiliations:** 1 Kunming Medical University, Kunming, China; 2 Affiliated Hospital of Yunnan University (The Second People’s Hospital of Yunnan Province), Kunming, China; 3 Dali University, Dali, China

**Keywords:** Leber’s congenital amaurosis, Variant, Crumbs homologue 1 (*CRB1*)

## Abstract

**Background:**

Leber’s congenital amaurosis (LCA) is a severe hereditary retinopathy disease that is characterized by early and severe reduction of vision, nystagmus, and sluggish or absent pupillary responses. To date, the pathogenesis of LCA remains unclear, and the majority of cases are caused by autosomal recessive inheritance. In this study, we explored the variant in the Crumbs homologue 1 (*CRB1*) gene in a Chinese family with LCA.

**Methods:**

We conducted comprehensive ocular examinations and collected 5 ml of blood samples from members of a Chinese family with LCA. A pathogenic variant was identified by capturing (the panel in NGS) and Sanger sequencing validation.

**Results:**

A nonsense variant (c.1499C>G) in the 6th exon of *CRB1* gene in a Chinese family with LCA was identified, which predicted a change in the protein p. S500*, may lead to loss of gene function. We summarized the 76 variants reported thus far in *CRB1* that caused LCA8.

**Conclusions:**

This study reported a novel variant c.1499C>G (p. S500*) of the *CRB1* gene occurred in a Chinese family with LCA, thus expanding the spectrum of *CRB1* variants causing LCA.

## Introduction

Since Theodore Leber first described Leber’s congenital amaurosis (LCA) in 1869, a great deal of information about LCA has been revealed, including both clinical characteristics and molecular genetics. LCA, a rare but important juvenile retinal dystrophy, is an inherited retinal disorder most often diagnosed in infancy in the first 6 months of life and characterized by the presence of nystagmus, poor visual acuity (VA), and a severely reduced or nondetectable electroretinogram [1, 2]. The global incidence of LCA ranges from 1**/**81,000 to 1**/**30,000 among newborn babies. Although the incidence is low, this disorder also causes blindness in 20% of LCA school-age children and accounts for approximately 5% of all cases of hereditary retinopathy [3, 4]. LCA is currently categorized into 21 types according to the pathogenic genes, with autosomal recessive inheritance as the dominant. LCA8 is caused by a homozygous or compound heterozygous variant in the *CRB1* gene (OMIM *604210) on chromosome 1q31.

## Material and methods

**Fig. 1:**
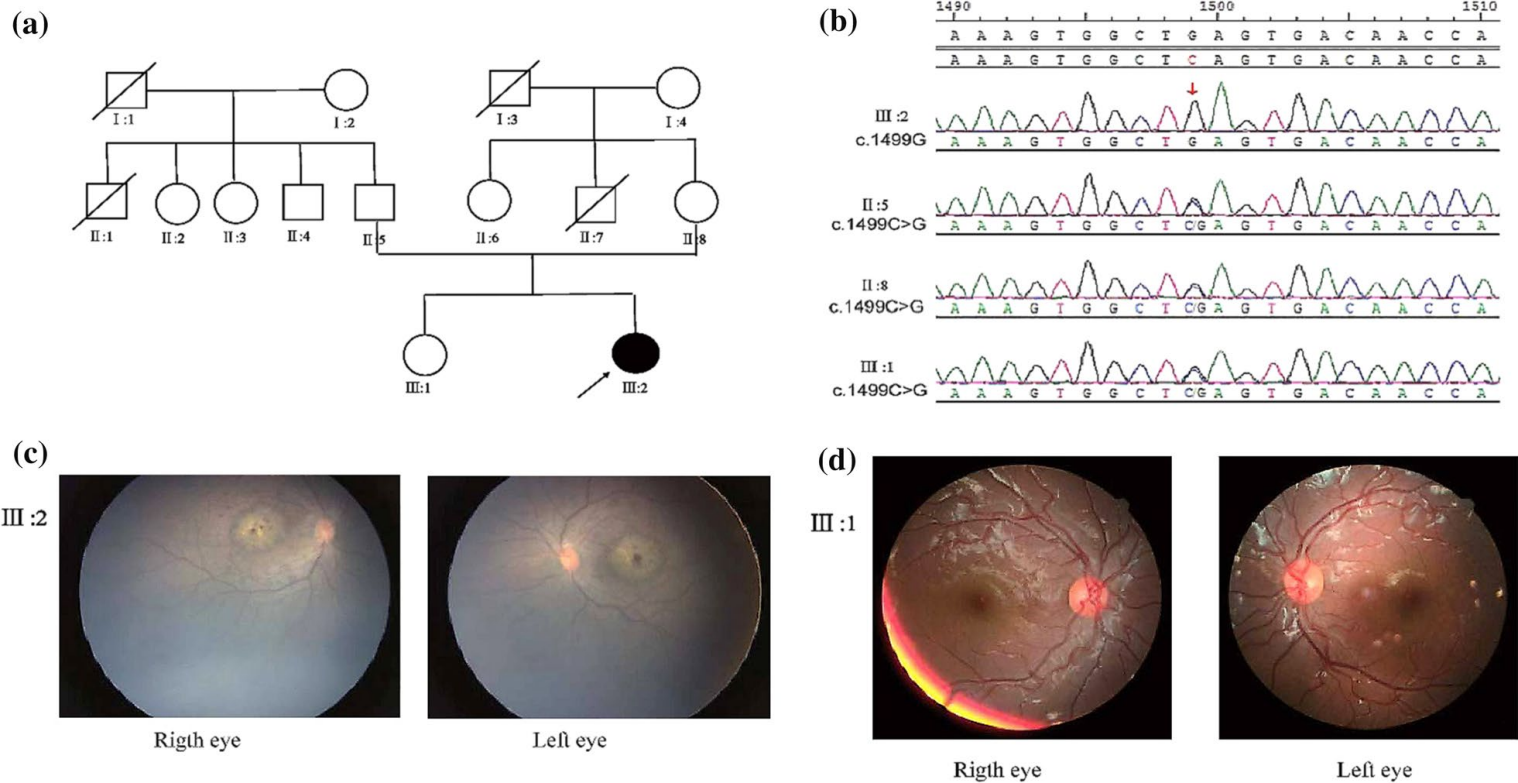
Pedigree of the LCA family with a *CRB1* variant, sequencing chromatogram, and diagnostic fundus. **a** Pedigree of the LCA family with a *CRB1* variant. The proband is marked by an arrow, black symbols denote affected members, white symbols denote unaffected members, squares denote males, and circles denote females. **b** Sequencing chromatograms. The affected proband showed a homozygous variant in the *CRB1* gene: nucleotide 1499 changed from a cytosine to guanine (c.1499C>G) homozygous variant, resulting in a nonsense variant of amino acids (p. S500*), her parents and sister show a heterozygous variant at the same site. **c** Diagnosis of the fundus. Both eyes of the proband showed pigmentation of the retina at the posterior pole that was peppery and salt-like, and the macular area was a mass of lesions with a large amount of pigmentation. **d** Her 5-year-old sister’s fundus is normal, and the same is true for her parents

The proband (Fig. 1), a 2-year-old girl, her parents complained that she has poor eyesight in both eyes and could not accurately grasp objects. She was unable to comply with the detailed eye examination. Under the guidance of the paediatrician, the opportunity for examination was obtained through oral anaesthesia. Her parents and sister underwent detailed eye examinations, including binocular corrected visual acuity, slit lamp examination, fundus photography, macular and optic disc OCT scanning, and electroretinogram (ERG).

Five millilitres of peripheral blood was obtained from each of the 4 subjects (II5, II8, III1, and III2) and collected in EDTA tubes for DNA extraction. The panel (463 genes related to ocular diseases) in next-generation sequencing (NGS) was used to capture the target gene. Then, Sanger sequencing was performed to validate the variants from 22 candidate genes. The transcript used to identify the variant in the CRB1 gene was NM_201253.

## Results

The proband's parents came from two unrelated families, with no consanguineous or inbreeding. Except for the proband, neither parent had a family member with similar eye disease (Fig. 1a).

After panel capture, 22 candidate genes remained. Primers were designed for each candidate gene, PCR was performed, and then, first-generation Sanger sequencing was used to verify the target gene. Finally, we obtained the target co-isolation gene in this family. Sequencing chromatograms: the proband shows a homozygous variant in the *CRB1* gene, nucleotide 1499 changed from cytosine (C) to guanine (G) c.1499C>G(p. S500*), her parents and sister showed a heterozygous variant at the same site (Fig. 1b).

The proband's eyes showed horizontal pendulum nystagmus. On examination, her eyes were in a normal position, the cornea and lens were clear, fundoscopy showed that the colour of the optic disc in both eyes was light, and the blood vessels from both eyes were thin and narrow (Fig. 1c). The pigmentation of the retina at the posterior pole was peppery and salt-like, and the macular area was a mass of lesions with much pigmentation.

After detailed eye examinations, the proband’s parents and sister showed normal results (Fig. 1d; Table 1).

**Table 1:** Clinical examination data

Patient	Gender	Age	Nucleotide amino acid	Homozygous/heterozygous	UCVA		CVA		Corneal optical refection	Nystagmus	Globe retraction
					OD	OS	OD	OS			
II:5	M	34	c.1499C>G (p.S500*)	Heterozygous	1.0	1.0	1.0	1.0	Normal	−	−
II:8	F	28	c.1499C>G (p.S500*)	Heterozygous	0.6	0.7	1.0	1.0	Normal	−	−
III:1	F	5	c.1499C>G (p.S500*)	Heterozygous	0.6	0.6	0.8	0.8	Normal	−	−
III:2	F	2	c.1499C>G (p.S500*)	Homozygous	unable	unable	unable	unable	Normal	+	−

Features of LCA8 and unaffected relatives

*UCVA* uncorrected visual acuity, *CVA* corrected visual acuity, *OD* right eye, *OS* left eye, +: positive, −: negative

For LCA, the criteria include signs of blindness or severe visual impairment from birth or within the first year of life, an ERG reduction of more than 50%, and congenital nystagmus [5]. Fundus examinations could reveal diagnostic clues, including peripheral pigmentary retinopathy, central maculopathy with or without bull’s eye pattern, or even macular atrophy. In addition, indispensable, molecular confirmation is needed.

In our study, the proband's eye examinations and genetic tests were consistent with the diagnosis of LCA. The homozygous variant in the 6th exon of *CRB1*: nucleotide 1499 changed from cytosine to guanine (c.1499C>G), resulting in a nonsense variant of amino acids (p. S500*), which has not been reported before. The proband's parents and sister had heterozygous variation at this site. According to the ACMG (American College of Medical Genetics and Genomics) guidelines, the variant was preliminarily determined to be pathogenic: PVS1 + PM2 + PM3_Supporting (hom). PVS1: This variant is a zero-effect variant (nonsense variant), which may lead to loss of gene function; PM2: This variant frequency in the database of the normal population (1000g2015aug_all, ESP6500si, GnomAD_Genome_ALL, GnomAD_Genome_EAS, etc.) is “-”, which means the variant was not detected; PM3_Supporting (HOM): This variant is a homozygous rare variant. No correlation of this locus was reported in the literature database. No pathogenicity analysis results were found in the ClinVar database. Our study expands the spectrum of *CRB1* variants causing LCA.

We used the ScanProsite tool (https://prosite.expasy.org/scanprosite/) to examine the secondary structure of the CRB1 protein. The nonsense variant c.1499C>G (p. S500*) is in *the laminin G domain profile* 485–670: score = 32.931.L, Yang, et al. also reported a nonsense variant c.1576C>T(p. R526X) in this domain [6]. *Laminin G* is an approximately 180 amino acid long domain found in a large and diverse set of extracellular proteins. It often occurs in multiple copies, probably serving as general protein interaction domains that bind target proteins and other macromolecules, such as carbohydrates. In most proteins, the precise function of the laminin G domain is unknown. A large number of ligands in the G domain of laminin have been reported, including heparin, sulfatides, integrins, dystroglycan, nidogen, and fibulin. In neurexin, the G domain is known to bind neurexophilins, α-latrotoxin and neuroligins [7, 8].

Another anatomical feature of LCA includes decreased thickness in different layers, especially in the outer nuclear layer (ONL), loss of integrity in the ellipsoid zone, and disorganized macular atrophy [9]. Unfortunately, the proband we reported was too young to cooperate with optical coherence tomography (OCT) and ERG examination, so we could not analyse the clinical features of these two aspects.

## LCA caused by *CRB1*

In 2004, Hanein et al. [10] reported a comprehensive mutational analysis of all known genes in 179 unrelated LCA patients, including 52 familial and 127 sporadic cases. The results showed that variants were identified in 47.5% of patients. *GUCY2D* appeared to account for most LCA cases in their series (21.2%), followed by *CRB1* (10%), RPE65 (6.1%), *RPGRIP1* (4.5%), *AIPL1* (3.4%), *TULP1* (1.7%), and *CRX* (0.6%). Three years later, Francesca Simonelli et al. [11] analysed 95 patients in Italy with LCA. They identified some novel variants that occurred frequently in the *RPE65* (8.4%), *CRB1* (7.4%), and *GUCY2D* (5.2%) genes. Through a detailed ophthalmic evaluation of patients with the variant, they found that *CRB1* variants were associated with reduced retinal thickness and a coarsely laminated retina (by OCT). In London, Henderson, R.H., et al. acquired DNA samples from 250 probands with LCA/early childhood-onset retinal dystrophy (EORD). They analysed using the LCA chip, and CRB1 variants were identified in 21 probands [12]. Corton et al. enrolled 404 Spanish patients in the study, 114 of which suffered from LCA and 290 from EORP (early-onset RP). Their study revealed that 11% of Spanish patients carried variants in *CRB1*, ranging from 9% of EORP to 14% of LCA cases. More than three-quarters of the variants identified were first described in their study [13].

Liping Yang et al. [6] used 18 cases presenting with LCA to identify disease-causing variants. They reported compound heterozygous variants of the *CRB1* gene, which included three novel heterozygous variants: c.3059delT (p. M1020Sfs*1), c.3460T>A (p. C1154S), and c.4207G>C (p.E1403Q). Hosono et al. reported variants of LCA- and inherited retinal dystrophy (IRD)-associated genes in 34 Japanese families, which is the first study to conduct a next-generation sequencing (NGS)-based molecular diagnosis of a large Japanese LCA cohort and achieved a detection rate of approximately 56%. Their results showed that the most frequently mutated genes were *CRB1*, *NMNAT1*, and *RPGRIP1 *[14]. Recently, Zhu et al. [15] enrolled 37 patients with strictly defined LCA in a cohort of IRD in ten years (2009–2019). Their results revealed that the *CRB1* gene occupied a greater proportion (27%) of associated LCAs in the western Chinese population.

*CRB1* variants are a common cause of LCA, and related variants include substitution, deletion, duplication and insertion. Table 2 lists the variants in LCA caused by *CRB1,* which include variant types, sites, corresponding amino acid changes and regions in recent years. These results are for readers' verification and reference.

**Table 2:** Summary of *CRB1* variants causing LCA8

Exon	Variant type	DNA change	Amino acid change	Region	References
Ex1	Substitution	c.2T>C	p.M1T	Japanese	Hosono et al. [14]
Ex1	Substitution	c.70+2T>A	–	Chinese	Zhu et al. [15]
Ex2	Substitution	c.107C>G	p.S36*	Pakistan	McKibbin et al. [16]
Ex2	Substitution	c.424G>T	p.G142*	uncertain	Beryozkin et al. [17]
Ex2	Substitution	c.471C>A	p.C157*	Chinese	Zhu et al. [15]
Ex2	Duplication	c.481dupG	p.A161Gfs*8	Spanish	Corton et al. [13]
Ex2	Deletion	c.498_506del	p.I167_G169del	England	Ahmed et al. [18]
Ex2	Deletion	c.613_619del	p.I205Dfs*13	Spanish	Corton et al. [13]
Ex2	Substitution	c.614T>C	p.I205T	England	Henderson et al. [12]
Ex3	Substitution	c.664G>A	p.E222K	Chinese	Li et al. [19]
Ex3	Duplication	c.668dupT	p.L223Ffs*4	Japanese	Hosono et al. [14]
Ex3	Duplication	c.733dupG	p.A245Gfs*16	Japanese	Hosono et al. [14]
Ex3	Substitution	c.866C>T	p.T289M	Italian	Simonelliet al. [11]
Ex3	Substitution	c.998G>A	p.G333D	Korea	Moon et al. [20]
Ex6	Deletion	c.1334_1740del	p.C445Yfs*8	Japanese	Hosono et al. [14]
Ex6	Substitution	c.1405T>G	p.C469G	Chinese	Zhu et al. [15]
Ex6	Substitution	c.1429G>A	p.G477R	Chinese	Yang et al. [6]
Ex6	Substitution	c.1499C>G	p.S500*	Chinese	this study
Ex6	Duplication	c.1567dupC	p.L523Pfs*28	Japanese	Hosono et al. [14]
Ex6	Substitution	c.1576C>T	p.R526*	Chinese	Yang et al. [6, 14]
Ex6	Substitution	c.1604T>C	p.L535P	Spanish	Corton et al. [13]
Ex6	Substitution	c.1678C>G	p.H560D	Chinese	Zhu et al. [15]
Ex6	Substitution	c.1690G>T	p.D564Y	Spanish	Corton et al. [13]
Ex6	Substitution	c.1750G>T	p.D584N	Uncertain	Hanein et al. [10]
Ex6	Substitution	c.1831T>C	p.S611P	Chinese	Yang et al. [6]
Ex6	Substitution	c.1841G>T	p.G614V	Chinese	Chen et al. [21]
Ex6	Deletion	c.1842delT	p.G614Gfs*6	Uncertain	Beryozkin et al. [17]
Ex6	Substitution	c.1903T>C	p.S635P	Chinese	Li et al. [19]
Ex6	Substitution	c.2107G>T	p.E703*	Iran	Saberi et al. [22]
Ex6	Substitution	c.2128G>C	p.E710Q	Uncertain	Hanein et al. [10]
Ex6	Substitution	c.2128+1G>A	-	Iran	Saberi et al. [22]
Ex7	Substitution	c.2222T>C	p.M741T	Uncertain	Hanein et al. [10]
Ex7	Deletion	c.2227delG	p.V743Sfs*11	Spanish	Corton et al. [13]
Ex7	Substitution	c.2234C>T	p.T745M	Chinese	Yang et al. [6]
Ex7	Deletion	c.2244_47del	p.S749del	Spanish	Corton et al. [13]
Ex7	Duplication	c.2276_2279dupCTTA	p.S758Sfs*33	Iran	Saberi et al. [22]
Ex7	Substitution	c.2290C>T	p.R764C	Uncertain	Hanein et al. [10]
Ex7	Substitution	c.2309G>T	p.G770V	Spanish	Corton et al. [13]
Ex7	Substitution	c.2401A>T	p.K801*	Italian	Simonelli et al. [11]
Ex7	Substitution	c.2479G>T	p.G827*	Uncertain	Hanein et al. [10]
Ex7	Substitution	c.2536G>T	p.G846*	Hungarian	Vamos et al. [23]
Ex7	Substitution	c.2548G>A	p.G850S	England	Henderson et al. [12]
Ex7	Substitution	c.2555T>C	p.I852T	Uncertain	Hanein et al. [10]
Ex7	Deletion	c.2676delG*	p.K892Nfs*95	England	Henderson et al. [12]
Ex8	Substitution	c.2677-2A>C	–	Chinese	Lin Li et al. [24]
Ex8	Deletion	c.2678-2682del	p.S893Sfs*14	Uncertain	Beryozkin et al. [17]
Ex8	Substitution	c.2688T>A	p.C896*	Spanish	Corton et al. [13]
Ex8	Substitution	c.2714G>A	p.R905Q	Chinese	Zhu et al. [15]
Ex9	Substitution	c.2843G>A	p.C948Y	Polish	Skorczyk et al. [25]
Ex9	Substitution	c.2843G>T	p.C948F	Uncertain	Hanein et al. [10]
Ex9	Insertion	c.2853_2854insT	p.A952fs*972	Uncertain	Hanein et al. [10]
Ex9	Substitution	c.2945C>A	p.T982K	Chinese	Zhu et al. [15]
Ex9	Substitution	c.3002T>A	p.I1001N	Spanish	Corton et al. [13]
Ex9	Substitution	c.3017C>A	p.S1006Y	Chinese	Zhu et al. [15]
Ex9	Substitution	c.3023T>G	p.L1008*	Chinese	Zhu et al. [15]
Ex9	Deletion	c.3059delT	p.M1020Sfs*1	Chinese	Yang et al. [6]
Ex9	Substitution	c.3068T>G	p.L1023R	Japanese	Hosono et al. [14]
Ex9	Substitution	c.3074G>T	p.S1025I	Uncertain	Hanein et al. [10]
Ex9	Substitution	c.3152 G>A	p.W1051*	Spanish	Corton et al. [13]
Ex9	Substitution	c.3218T>A	p.L1073Q	Chinese	Zhu et al. [15]
Ex9	Substitution	c.3221T>C	p.L1074S	Chinese	Lin Li et al.[16]
Ex9	Substitution	c.3290T>A	p.L1097Q	Chinese	Zhu et al. [15]
Ex9	Substitution	c.3299T>C	p.I1100T	Spanish	Corton et al. [13]
Ex9	Substitution	c.3307G>A	p.G1103R	Italian	Simonelli et al. [11]
Ex9	Substitution	c.3320T>G	p.L1107R	Uncertain	Hanein et al. [10]
Ex9	Deletion	c.3345delT	p.G1115fs*1140	Uncertain	Hanein et al. [10]
Ex9	Substitution	c.3466G>T	p.D1156Y	Uncertain	Hollanderet al. [26]
Ex9	Substitution	c.3482A>G	p.Y1161C	Spanish	Corton et al. [13]
Ex9	Duplication	c.3542dupG	p.C1181Wfs*12	England	Henderson et al. [12]
Ex11	Substitution	c.3879G>A	p.W1293*	Uncertain	Hanein et al. [10]
Ex11	Substitution	c.3961T>A	p.C1321G	Uncertain	Hanein et al. [10]
Ex11	Deletion	c.3988delG	p.E1330fs*1340	Uncertain	Hanein et al. [10]
Ex11	Deletion	c.4000delG	p.V1334W fs*7	Spanish	Corton et al. [13]
Ex12	Substitution	c.4005+1G>A	–	Chinese	Zhu et al. [15]
Ex12	Substitution	c.4013+1G>T	–	Uncertain	Hollanderet al. [21]
Ex12	Deletion	c.4121_4130del	p.R1374fs*1397	Uncertain	Hanein et al. [10]

“–”: not applicable

To date, a total of 76 *CRB1* variants have caused LCA. Furthermore, it has been reported that variants in *CRB1* are responsible for 7.4%-27% of LCA in different populations. The pathogenic variants were mainly substitution and deletion, including duplication, insertion, which reflected the richness of variant types (Table 3). The variant sites of LCA8 were mainly concentrated in exons 6, 7 and 9 of *CRB1*, and clear pathogenic sites were found in all exons except exons 4, 5 and 10, indicating the universality of variant regions (Table 4). The reported cases involved more than 10 countries and regions, including China, England, Japan, Spain and Italy, which also showed that the global coverage of LCA caused by *CRB1* is extensive.

**Table 3:** Types and proportion of *CRB1* variants causing LCA8

Types of variants	Substitution	Deletion	Duplication	Insertion
Count	56	13	6	1
Percentage	73.7%	17.1%	7.9%	1.3%

**Table 4:** Numbers and proportion of *CRB1* exon variants causing LCA8

Exon	Ex1	Ex2	Ex3	Ex4	Ex5	Ex 6	Ex 7	Ex8	Ex9	Ex10	Ex11	Ex12
Count	2	7	5	0	0	17	13	4	21	0	4	3
Percentage	2.6%	9.2%	6.6%	0	0	22.4%	17.1%	5.3%	27.6%	0	5.3%	3.9%

## Other diseases of retinal dystrophy caused by *CRB1* variants

In addition to LCA, variants in *CRB1* are associated with several other diseases of retinal dystrophy: Rosa Riveiro-Alvarez, et al. [27] reported an early-onset RP phenotype in a Spanish family caused by the Nonsense *CRB1* c.2843G>A(p. C948Y) variant. Two *CRB1* substitution variants, c.3991C>T(p. R1331C) and c.4142C > T(p. P1381 L), were reported to illustrate a novel presentation of macular dystrophy caused by *CRB1* variants by Stephen H. Tsang et al. [28]. Arif O. Khan et al. uncovered a homozygous *CRB1* variant c.80G>T(p. C27F) in three siblings with childhood cone-rod dystrophy and macular cystic degeneration in a family [29]. Ajoy Vincent et al. reported biallelic variants (p. G123C and p. C948Y, p. I167_G169del and p. R764C) in *CRB1* in two families caused autosomal recessive familial foveal retinoschisis, which may be the mildest end of the spectrum of *CRB1*-related diseases [30]. Benjamin K. Ghiam et al. reported that a novel variant c.4014T>A in *CRB1* was related to retinal degeneration and may portend a poor prognosis for CME responsiveness to therapy [31].

## Discussion

LCA is the earliest and most severe hereditary retinopathy, in which the function of cone-rod cells in both eyes is completely lost at birth or within one year after birth, leading to congenital blindness in infants. The majority of cases are caused by autosomal recessive inheritance. Typical characteristics of LCA include early and severe reduction of vision associated with nystagmus, photophobia, sluggish or absent pupillary responses, finger pressure on eyeballs, fundus appearance, ranging from normal, maculopathy, to typical RP-like abnormalities, and electroretinogram showed that A and B waves were flat and even severely reduced to nondetectable. It can also be accompanied by keratoconus, hyperopia, developmental delay and nervous system abnormalities [32].

In some cases/reports, there are many similar clinical features between LCA and early-onset RP, and even the diagnosis is ambiguous[33]. Early-onset RP is usually considered to be a relatively milder form, in which patients do not have a congenital onset of visual impairment. We could distinguish the following phenotypes: LCA, early onset retinal degeneration; RP, presence of preservation of the para-arteriolar retinal pigment epithelium and Coats-like vasculopathy[34].

To date, 28 genes involved in the pathogenesis of LCA [35]. *CRB1* belongs to LCA8. The *CRB1* gene maps to chromosome 1q31.3 and is composed of 12 exons; the longest isoform consists of 1,406 amino acids. This gene encodes a protein that is similar to the Drosophila crumb protein and localizes to the inner segment of mammalian photoreceptors. In Drosophila, crumbs localize to the stalk of the fly photoreceptor and may be a component of the molecular scaffold that controls proper development of polarity in the eye[36], and *CRB1* has been found to be important in maintaining cellular polarity[37].

In the mouse retina, *CRB1* is expressed in the inner segment of the photoreceptors and Muller cells to maintain adequate morphogenesis and polarity in retinal development[38]. Therefore, *CRB1* gene variants often lead to a variety of retinal dystopathies, including retinitis pigmentosa (RP), LCA, and macular dystrophy. Approximately 9–17% of LCA cases have been related to *CRB1* variants, which is especially higher in the Chinese population[39, 40]. A wide variety of visual acuity was noted in patients with variants in *CRB1*, ranging from 20/30 to NLP[10, 41].

Among LCA, *RPE65* variants were almost always associated with normal macular thickness, as assessed by OCT, whereas *CRB1* variants were associated with reduced retinal thickness and a coarsely laminated retina. Fundus abnormalities were more heterogeneous in carriers of *CRB1* variants. In fact, some scholars observed salt-and-pepper retinal dystrophy in younger patients and subsequently massive spicular and not nummular pigmentation at the posterior pole, which was reported to be a phenotypic feature of carriers of *CRB1* variants[11]. Saloni Walia et al. [42]. Through a multicentre retrospective observational study with 169 patients with LCA, variants in *RPE65* (LCA-Type Ii) and *CRB1* (LCA-8) may be associated with a relatively better VA in early life compared with other gene variants. The onset of the symptoms of LCA after the age of 1 year is also associated with an overall better VA prognosis.

## Conclusions

LCA is one of the earliest and most severe forms of inherited IRD. Patients suffer from severe visual impairment during childhood, with their vision continuously deteriorating, the final outcome of which is usually complete loss of vision by their thirties or forties[43]. Therefore, it is very important to find an effective treatment. Albert et al. provided an entirely new dimension in ocular therapeutics for gene therapy to LCA2. Patients with LCA2 who received AAV2.hRPE65v2 by subretinal injection showed evidence of improvement in retinal function, pupillary light reflex, and reduction in nystagmus. These clinical trials are approaches to the treatment of LCA and possibly other forms of retinal degeneration[44].

However, much is still unknown about the pathogenesis of LCA. With the improvement of next-generation sequencing technology and the application of various molecular biological means, research on corresponding cell functions, the identification of gene subtypes and the establishment of animal models have greatly promoted our understanding of LCA. These latest advances provide a steady stream of evidence for a better understanding and treatment of LCA in the future. These findings may be useful for faster gene diagnosis, prenatal testing, the development of potential gene therapies, and for improving the understanding of the molecular pathogenesis of LCA.

## Data Availability

The relevant data were generated during this study and included in this article. Raw sequence data were not available in this article, as no datasets were generated during the current study. The corresponding author Liwei Zhang (drzhangliwei@163.com) should be contacted if someone wants to request the data from this study.
